# Prediction Model of Organic Molecular Absorption Energies based on Deep Learning trained by Chaos-enhanced Accelerated Evolutionary algorithm

**DOI:** 10.1038/s41598-019-53206-1

**Published:** 2019-11-21

**Authors:** Mengshan Li, Suyun Lian, Fan Wang, Yanying Zhou, Bingsheng Chen, Lixin Guan, Yan Wu

**Affiliations:** College of Physics and Electronic Information, Gannan Normal University, Ganzhou, Jiangxi 341000 China

**Keywords:** Chemistry, Computer science

## Abstract

As an important physical property of molecules, absorption energy can characterize the electronic property and structural information of molecules. Moreover, the accurate calculation of molecular absorption energies is highly valuable. Present linear and nonlinear methods hold low calculation accuracies due to great errors, especially irregular complicated molecular systems for structures. Thus, developing a prediction model for molecular absorption energies with enhanced accuracy, efficiency, and stability is highly beneficial. By combining deep learning and intelligence algorithms, we propose a prediction model based on the chaos-enhanced accelerated particle swarm optimization algorithm and deep artificial neural network (CAPSO BP DNN) that possesses a seven-layer 8-4-4-4-4-4-1 structure. Eight parameters related to molecular absorption energies are selected as inputs, such as a theoretical calculating value E_c_ of absorption energy (B3LYP/STO-3G), molecular electron number N_e_, oscillator strength O_s_, number of double bonds N_db_, total number of atoms N_a_, number of hydrogen atoms N_h_, number of carbon atoms N_c_, and number of nitrogen atoms N_N_; and one parameter representing the molecular absorption energy is regarded as the output. A prediction experiment on organic molecular absorption energies indicates that CAPSO BP DNN exhibits a favourable predictive effect, accuracy, and correlation. The tested absolute average relative error, predicted root-mean-square error, and square correlation coefficient are 0.033, 0.0153, and 0.9957, respectively. Relative to other prediction models, the CAPSO BP DNN model exhibits a good comprehensive prediction performance and can provide references for other materials, chemistry and physics fields, such as nonlinear prediction of chemical and physical properties, QSAR/QAPR and chemical information modelling, etc.

## Introduction

As an important physical property of molecules, the absorption energy contains internal structural information and electronic performance of molecules. The accurate prediction of absorption energies is an important direction in the field of computational chemistry with great research value and significance^1,2^. Many linear and nonlinear computational methods such as linear regression, density functional theory, support vector machine, and artificial neural network have been applied to examine the absorption energies of organic molecules^3–5^.

Hutchison *et al*.^6^ used ZINDO/CIS, ZINDO/RPA, HF/CIS, HF/RPA, TDDFT/TDA, and TDDFT to predict the absorption energies of 60 organic molecules and identified that the linear regression achieved superior combined performances for TDDFT/CIS and TDDFT/RPA. However, for complicated molecules or a large system, these kinds of methods fall short in performance. Gao *et al*.^7,8^ used the least squares support vector machine to reduce the errors of absorption energies of 160 organic molecules, the multiple linear regression method to seek for characteristic space and select the main molecular physical parameters, and the least squares support vector machine to establish a nonlinear model. Results showed that the least squares support vector machine was a more accurate and effective correction method in the field of physical chemistry than the other methods. Li *et al*.^9^ obtained the absorption energies of 60 molecules by calculating through TDDFT//B3LYP, corrected using the artificial neural network and multiple linear regression, and found that the artificial neural network was better than multiple linear regression. However, the initial weight of the artificial neural network was obtained randomly; such initial weight usually results in slow convergence and low performance and be caught potentially in the local minimum to cause a poor prediction effect. To improve the deficiencies of the artificial neural network, scholars used various intelligence algorithms, such as simulated annealing algorithm, genetic algorithm, particle swarm optimization (PSO) algorithm, and ant colony algorithm, to optimize the parameters of artificial neural network and have successfully improved the prediction accuracy of absorption energies^10–12^. Gao *et al*.^13^ used the GANN method was utilized to correct the absorption energies of 150 molecules, compared GANN and BP artificial neural network, and observed that the GANN method was obviously superior to the BP neural network method in predicting absorption energies.

Deep learning has attracted much concern from the academic circles and industrial circles because of its powerful learning ability in recent years^14–23^. Deep learning is effective at digging the abstracter and abstracter feature representation from original input data, and the representation achieves a favorable generalization ability and has overcome some problems considered difficult to solve in past artificial intelligence^24–28^. Moreover, with significant growth of quantity of training datasets and strengthened chip processing ability, deep learning held outstanding achievements in the fields of artificial intelligence and computational chemistry, and deep neural network (DNN) is the main deep learning form^29,30^. However, for the data-driven model, excessively redundant input variables not only lead to excessive training time but also increase the overfitting risk, particularly, for DNNs with many parameters.

Given the problems presently existing in the DNN, establishing an absorption energy prediction model with improved accuracy, efficiency, and stability is expected. Therefore, with the accelerated PSO algorithm reported in recent years, this paper proposes and discusses a chaos-enhanced accelerated PSO (CAPSO) algorithm and then uses this enhanced algorithm to train a DNN and formulate a deep learning model based on the swarm intelligence algorithm. Subsequently, this model is applied to predict the absorption energies of organic molecules.

## Model Theory

### DNN

**Figure 1:**
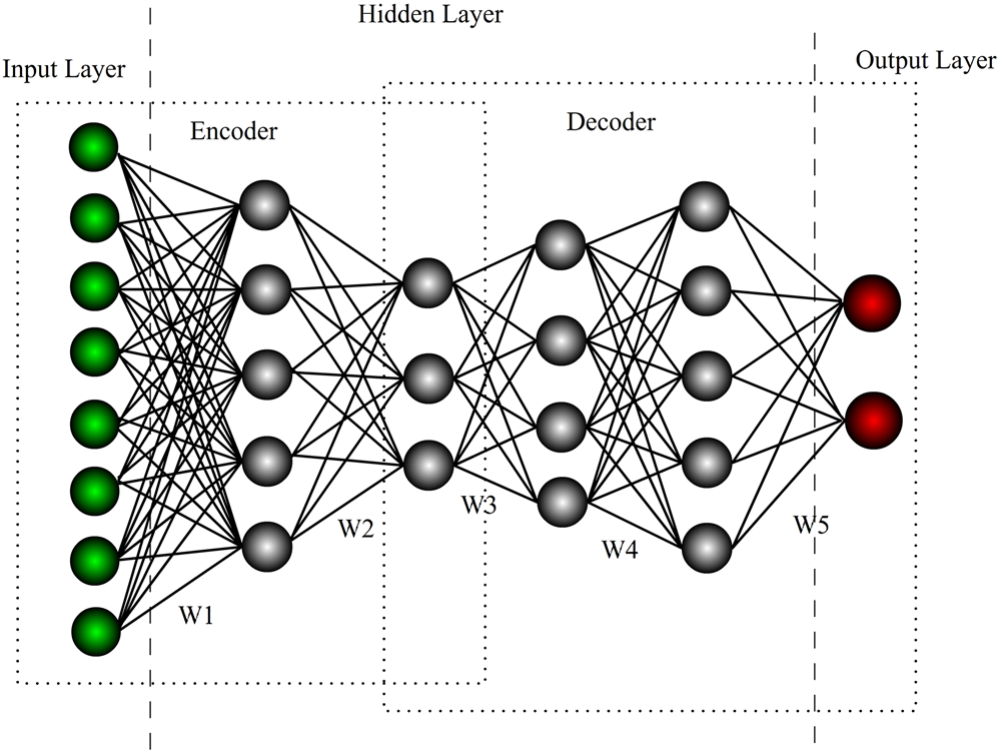
Deep neural network model.

As a brand-new field that has witnessed rapid development over 10 years, deep learning has attracted attention from an increasing number of researchers^31,32^. Deep learning is a hierarchical machine learning method containing multistage nonlinear transformation. DNN is the present main form, and its structure is shown in Fig. 1.

DNN is a multilayer perceptron but contains multiple hidden layers. Figure 1 shows a typical forward-direction DNN model with five hidden layers. In this structure, connection does not exist between nodes belonging to the same layer, but nodes at neighboring layers are mutually connected. Sigmoid function is generally adopted for the excitation function of nodes at hidden layers, whereas the softmax function is generally used for nodes at the output layer.

In a *L* + 2-layer DNN model with an input variable of *o* containing one input layer, one output layer, and *L* hidden layers. For one hidden layer $l,(l=0,\,\mathrm{...},\,L-1)$, the output vector ${h}_{j}^{L}$ is as follows:

$${h}_{j}^{l}=\delta ({z}_{j}^{l}({v}^{l}))=\delta ({({\omega }_{j}^{l})}^{T}{v}^{l}+{\alpha }_{j}^{l}),j=1,\,\mathrm{...},\,{N}^{l}$$

where $\delta ({z}_{j})=\frac{1}{1+{e}^{-{z}_{j}}}$ is the sigmoid function; ${\omega }_{j}^{l}$ and ${\alpha }_{j}^{l}$ are the weight and deviation of the j (th) node at the hidden layer *l* respectively; and *v*^*l*^ is the input vector of the hidden layer *l*. When *l* = 0, *v*^*l*^ is the input vector *o*, or otherwise *v*^*l*^ = *h*^*l*−1^.

For an output layer *L*, the output vector ${h}_{j}^{L}$ is

$$\begin{array}{rcl}{h}_{j}^{L} & = & soft\,{{\rm{\max }}}_{j}({z}^{L}({v}^{L})),j=1,\,\mathrm{...},\,{N}^{L}\\ {z}^{L}({v}^{L}) & = & {({\omega }^{L})}^{T}{v}^{L}+{a}^{L}\end{array}$$

where *v*^*l*^ = *h*^*l*−1^ is the input at the output layer, namely, the output of hidden layer *L* − 1 and *ω*^*L*^, *α*^*L*^ and *N*^*L*^ are weight, deviation, and number of nodes, respectively, of the output layer.

DNN training aims at optimizing the weights and deviations of the hidden layers and input layer, namely, optimizing parameters ${\omega }_{j}^{l},{\alpha }_{j}^{l}$, $j=1,\,\mathrm{...},\,{N}^{L}$ and $l,(l=0,\,\mathrm{...},\,L)$. DNN can be rapidly and effectively trained and a critical factor in acquiring excellent performance. Therefore, this paper proposes and discusses a rapid and highly efficient improved PSO algorithm, abbreviated as CAPSO algorithm used for DNN training.

### Chaos-enhanced accelerated particle swarm algorithm

The PSO algorithm is a kind of swarm intelligence algorithm proposed by scholars Eberhart and Kennedy^33–35^, but the standard PSO algorithm holds deficiencies, such as sensitivity to parameters, premature convergence, and slow local search. A variant called accelerated PSO algorithm (APSO) has been a cause of concern by scholars in recent years. However, while improving the convergence speed, the APSO algorithm also harbors a premature convergence problem and will possibly miss some extreme values. Therefore, the chaos theory is blended into the optimization of the APSO algorithm and a new CAPSO algorithm is proposed.

In the APSO algorithm, the location updating formula is shown below:

1 $${{\rm{x}}}_{{\rm{i}},{\rm{d}}}^{{\rm{k}}+{\rm{1}}}=(1\,-\,{{\rm{C}}}_{2}){{\rm{x}}}_{{\rm{i}},{\rm{d}}}^{{\rm{k}}}+{{\rm{C}}}_{2}{{p}}_{{\rm{g}},{\rm{d}}}^{{\rm{k}}}+{C}_{1}r$$

where *C*_1_ and *C*_2_ are learning factors; r is a random number between (0, 1); ${{\rm{x}}}_{{\rm{i}},{\rm{d}}}^{{\rm{k}}}$ is the d-dimensional location in the k (th) iteration; and ${{\rm{p}}}_{{\rm{g}},{\rm{d}}}^{{\rm{k}}}$ is the location of the d-dimensional global extremum.

Compared with the standard PSO algorithm, two parameters, namely, *C*_1_ and *C*_2_ were used in APSO. To reduce the randomness in the iteration process, *C*_1_ was expressed as a monotone decreasing function, namely, ${C}_{1}={\delta }^{t}$, where $0 < \delta  < 1$ and *t* is the present number of iterations. Therefore, the APSO performance was mainly influenced by parameter *C*_2_. The classical logistic equation was utilized to realize the evolution of the chaos variable and parameter optimization, and the iterative formula is as follows:

2 $${{\rm{X}}}_{i}^{k+1}=4{{\rm{x}}}_{i}^{k}(1-{{\rm{x}}}_{i}^{k})$$

The influences of the inertia weight factor and cognitive factor on particles will not be considered in the CAPSO algorithm, and particles are only constrained by the global extremum during the whole searching process, which accelerates searching while guaranteeing the searching accuracy. Table 1 shows the details of the hyper-parameters in CAPSO algorithm.

**Table 1:** Details of the hyper-parameters in CAPSO algorithm.

Parameter	Description	Value
m	Number of particles	60
itmax	Iteration times	2000
minerror	Minimum error	1.00E-07
c_1_	Cognitive component	Generated by *C*_1_ = *δ*^*t*^
c_2_	Social component	Generated by logistic equation

### CAPSO algorithm-based DNN model

The most commonly used DNN is an artificial neural network based on multilayer error back propagation (BP ANN)^36^, abbreviated as BP DNN and consists of an input layer, several hidden layers, and an output layer, and a fully connecting structure is realized between the layers. In the BP DNN, the model determines the weight and deviation between network layers so s to establish a nonlinear relation between the input and output. This nonlinear relation between input and output can be understood from structural analysis as input

$$y=f({w}_{l}^{j}{a}_{l}^{j}),\,j=1,\ldots ,{N}^{L};\,l=0,\ldots ,L$$

where ${\omega }_{j}^{l}$ and ${\alpha }_{j}^{l}$ are the weight and deviation of the j (th) node at layer *l*, respectively, and the network performance was mainly decided by the parameter ${\omega }_{j}^{l},{\alpha }_{j}^{l}$.

In this paper, the PSO algorithm was used to optimize two key parameters ${\omega }_{j}^{l},{\alpha }_{j}^{l}$ of BP DNN, and CAPSO algorithm-based BP DNN model was obtained and abbreviated as CAPSO BP DNN; therefore, in the CAPSO optimization algorithm, particles are designed as a structure containing weight vector ${\omega }_{j}^{l}$ and deviation vector ${\alpha }_{j}^{l}$, namely

3 $$particle(i)=[{\omega }_{j}^{l},{\alpha }_{j}^{l}]$$

### Performance evaluation of the model

Model evaluation is implemented mainly in two aspects—model stability and reliability. For a general calculation model, assessment is conducted in terms of prediction accuracy, efficiency, and stability. The indexes reflecting the prediction accuracy are absolute average relative error (*AARD)* and root-mean-square error of prediction (*RMSEP*), which are defined as follows:

4 $$AARD=\frac{1}{N}\mathop{\sum }\limits_{i=1}^{N}\frac{|{\overline{y}}_{i}-{y}_{i}|}{{y}_{i}}$$

5 $$RMSEP=\sqrt{\frac{1}{{\rm{N}}}\mathop{\sum }\limits_{{\rm{i}}=1}^{{\rm{N}}}({y}_{i}-{\bar{y}}_{i}{)}^{2}}$$

The index reflecting the correlation between the predicted value and experimental value is the average correlation coefficient (*R*^2^), which is defined as follows:

6 $${R}^{2}=\frac{{[\mathop{\sum }\limits_{{\rm{i}}=1}^{{\rm{N}}}({y}_{i}-{y}_{ave})({\bar{y}}_{i}-{\bar{y}}_{ave})]}^{2}}{\mathop{\sum }\limits_{{\rm{i}}=1}^{{\rm{N}}}({y}_{i}-{y}_{ave}{)}^{2}\mathop{\sum }\limits_{{\rm{i}}=1}^{{\rm{N}}}({\bar{y}}_{i}-{\bar{y}}_{ave}{)}^{2}}$$

In these formulas, N is the number of samples, ${\bar{y}}_{i}$ is the model predicted value of calculated value, *y*_*i*_ is the experimental actual value, *y*_*ave*_ is the average of the actual value of the sample, and ${\bar{y}}_{ave}$ is the average of the predicted values.

## Results and Discussion

### Experimental datasets

Experimental data related to the absorption energies of 160 organic molecules are totally collected in this paper. To obtain a prediction model with improved generalization ability, an experimental database is divided into five groups by magnitude of absorption energies. About 70% data are extracted using the random selection method from each group to the training set; then, 15% of the data are extracted to the verification set and test set, and statistical data are obtained in Table 2.

**Table 2:** Statistical table of experimental data.

No	Absorption Energies (eV)	Data points	Training	Validation	Testing	References
1	2.69–2.99	12	8	2	2	^7,13^
2	3.01–3.96	30	22	4	4	^7,13^
3	4.00–4.68	33	23	5	5	^7,8^
4	4.70–5.08	39	27	6	6	^7,8,13^
5	5.10–6.66	46	32	7	7	^7,8,13^
	Total	160	112	24	24	

### Model structure

**Figure 2:**
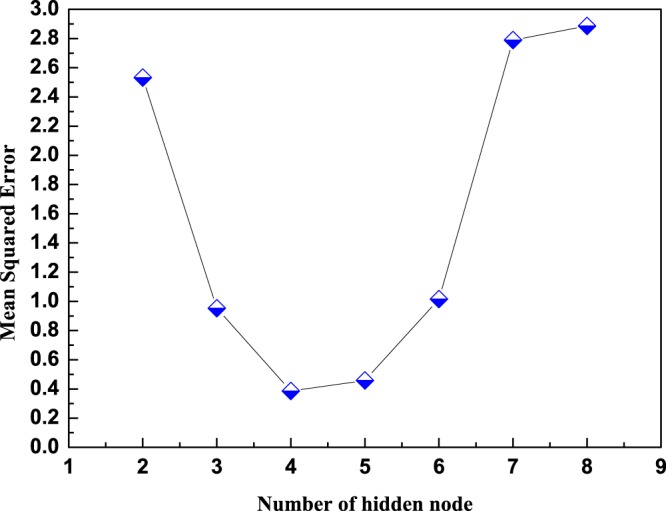
Comparison diagram of the optimization of numbers of nodes at hidden layers.

The number of nodes at the input layer of the model is determined by the influencing factors in the research on practical problems. Chen *et al*.^37^ used three descriptors including a theoretical calculating value of absorption energy(E_c_), molecular electron number (N_e_) and the oscillator strength (O_s_), and predicted the adsorption energy of molecules. Gao *et al*.^13^ used six descriptors including E_c_, N_e_, O_s_, number of double bonds (N_db_), total number of atoms (N_a_), and the correlated dipole moment (D_m_), and the computational results are promising. In this paper, in order to obtain a further accurate computational efficiency, we selected eight parameters that quite related to molecular absorption energies for developing a better performance model. These parameters include E_c_, N_e_, O_s_, N_db_, N_a_, the number of hydrogen atoms (N_h_), the number of carbon atoms (N_c_), and the number of nitrogen atoms (N_N_). Therefore, eight input parameters exist in the CAPSO BP DNN model. The number of nodes at the input layer was 8. One node represents the molecular absorption energy at the output layer, namely, one output parameter. The number of hidden layers commonly used a five-layer structure; all hidden layers achieved the same number of nodes, and the numbers of nodes achieved from the hidden layers were 2 to 8 for trial. Figure 2 shows a comparative relation scheme of the prediction errors and the numbers of nodes at hidden layers.

The comparison diagram shows that the Mean Squared Error (MSE) initially progressively decreased and then increased with increasing numbers of nodes at the hidden layers. When the number of nodes was large, the error growth obviously accelerated, when number of nodes was 7, the training error abruptly increased. When the number of nodes was 4, the training MSE was minimum, and the structure of the prediction model was optimal at the time, namely, the model structure was 8-4-4-4-4-4-1.

### Results and analysis

#### Analysis of the experimental results of the proposed model in this paper

**Figure 3:**
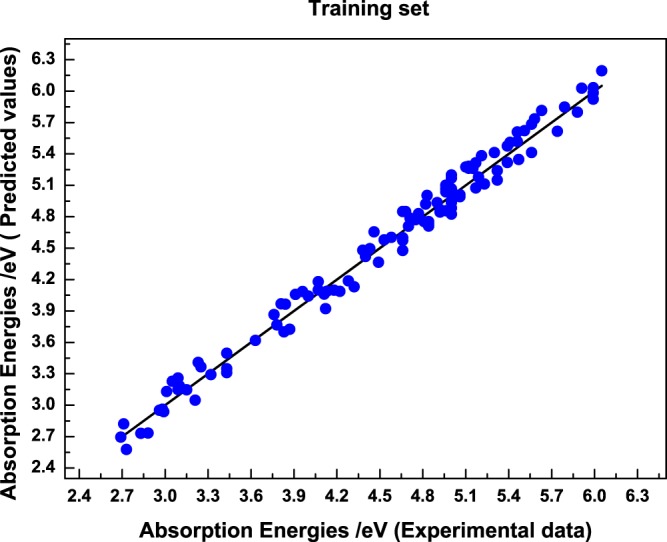
Comparison diagram between the predicted value and actual value in the training set.

**Figure 4:**
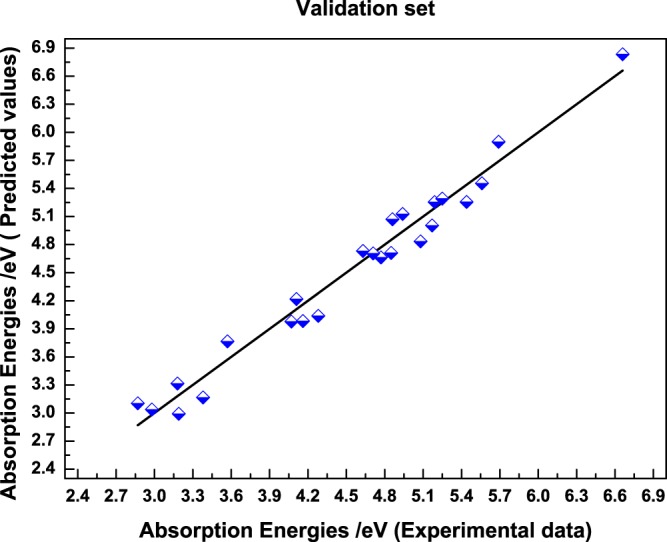
Comparison diagram between the predicted value and actual value in the verification set.

A seven-layer 8-4-4-4-4-4-1 CAPSO BP DNN prediction model was established and used to predict the molecular absorption energies. Initially, 112 groups and 24 groups of data in the training set and verification set were respectively used for model training and verification. Figures 3 and 4 show comparison diagrams between the actual value and model predicted value of the molecular absorption energies in the training set and verification set, respectively. As shown in the figures, a straight line represents an ideal model, in which the predicted value is equal to experimental value. A circle and rhombus represent model predicted values in a training set and verification set. The perpendicular distance between the data point of the predicted value and straight line expresses the absolute error between predicted value and experimental value.

**Figure 5:**
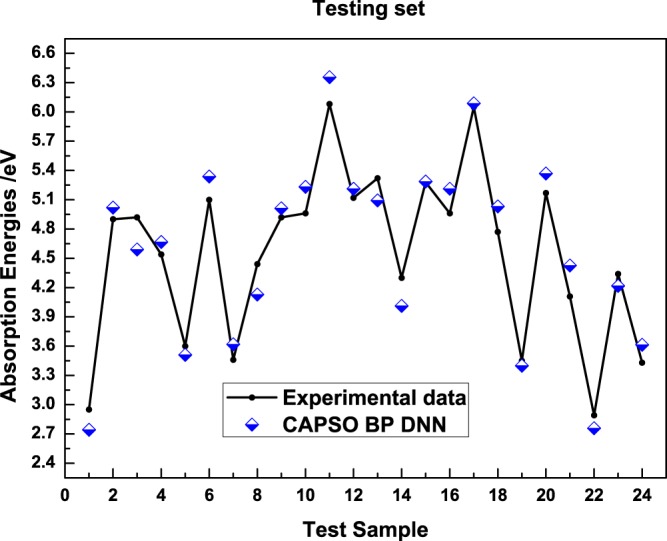
Comparison diagram between the predicted value and actual value of sample absorption energies in the test set.

The figures show that the model predicted values are distributed near the actual values regardless of the training set and verification set and are highly consistent with the experimental values. On the basis of the perpendicular distance between the prediction point and straight line, model prediction error is small with a high prediction accuracy. Figure 5 is a relational graph between the actual value and model predicted value of the absorption energies in the test set.

In the testing results, the model predicted value was highly consistent with the actual value in the test set; this finding indicates that the model holds a favorable prediction ability. Table 3 shows the experimental statistical data of the model in the training set, verification sets and test set.

**Table 3:** Statistical data of the model prediction performance.

Subset	*AARD*	*R*^2^	*RMSEP*
Training	0.026	0.9972	0.0146
Validation	0.028	0.9969	0.0148
Testing	0.033	0.9957	0.0153
Average	0.029	0.9966	0.0149

In the statistical data of three subsets, the model applies a good prediction effect in the subsets with small prediction error and optimal comprehensiveness. The table suggests that the good prediction performance of the model is reflected by the prediction accuracy and correlation. The above results can verify that model prediction performance was outstanding.

#### Results analysis of the models of comparison

**Figure 6:**
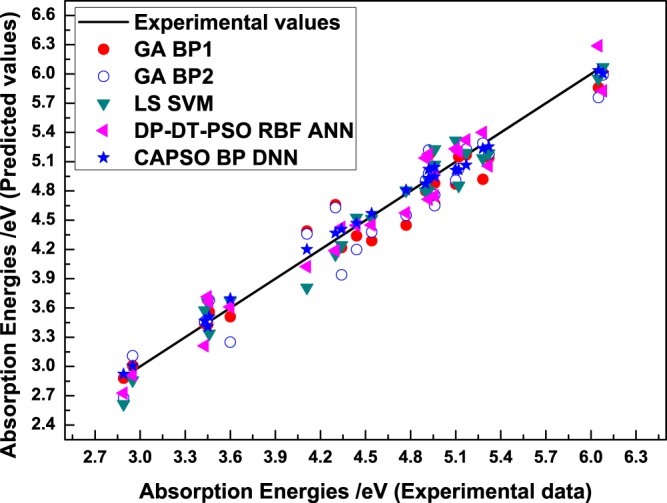
Comparison of the test results of the models.

To verify the comprehensive performance of the deep learning-based CAPSO BP DNN model, we selected GABP1^13^, GABP2^13^, (LS SVM)^7,8^ and DP-DT-PSO RBF ANN^38^ from literature reports as models of comparison, and the model details refer to relevant literature. Figure 6 displays the prediction results of the models in the test set.

**Figure 7:**
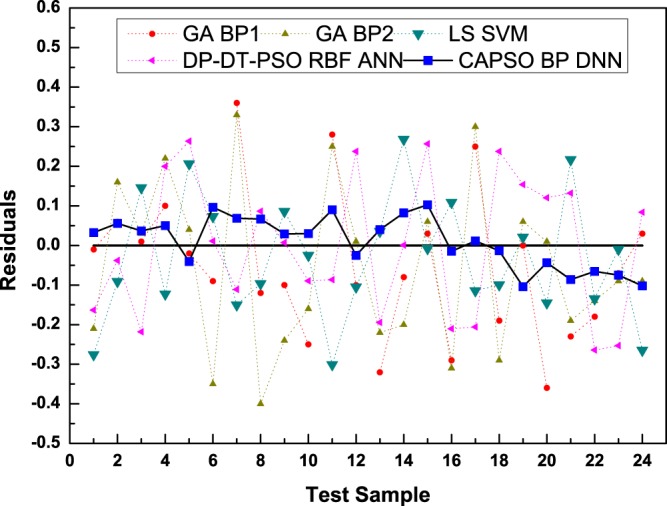
Comparison of the residual error curves of the test results of the models.

On the basis of the perpendicular distance between the prediction point and straight line, the predicted data of the CAPSO BP DNN model all distribute near the experimental value with small prediction error, and the comprehensive prediction performance was obviously superior to those of other methods. Figure 7 reveals the residual error curves between the experimental value and predicted value of the models in the test set.

The error curve graphs suggest that GA BPA performs almost equally well as GA BP2; the performance of the LS SVM was basically equivalent to that of DP-DT-PSO RBF ANN, whereas DP-DT-PSO RBF ANN showed a minor dominance. Most errors of the CAPSO BP DNN model proposed in this paper were distributed within [−0.1, 0.1]. The errors at individual prediction points were large; most were near 0. Thus, the performance was obviously superior to those of the other models of comparison. Table 4 calculates the evaluation results of the models.

**Table 4:** Statistical results of the comparative models.

Model	*AARD*	*R*^2^	*RMSEP*	*CT*^*a*^
GA BP1	0.1685	0.9168	0.1572	63
GA BP2	0.1637	0.9137	0.1566	65
LS SVM	0.1345	0.9324	0.1184	48
DP-DT-PSO RBF ANN	0.0754	0.9813	0.0563	86
CAPSO BP DNN	0.0330	0.9957	0.0153	76

^a^Convergence time (s).

The accurate calculation data in the table reveal that the accuracy of the correlation of the CAPSO BP DNN model was obviously dominant, and the correlation was above 99%. The main reason for the dominance of the model proposed in this paper was the introduction of the chaos-accelerated mechanism into the PSO evolutionary algorithm. This modification improved the model training and prediction performance. For the convergence time *CT*, the convergence time of the CAPSO BP DNN model was moderate. The general DNNs consumed a long period, but the accelerated mechanism in the CAPSO BP DNN lasted for a shortened time.

#### Result discussion

In this paper, we confirm the performance of the proposed computational model from the accuracy and correlation, compared with the other models, this model has the following characteristics and deficiencies:

1. The eight input parameters of the model can more accurately calculate the absorption energy of the molecule.
  The model uses a 7-layer deep neural network modeling, and eight attributes closely related to the molecular absorption energy were selected as the input variables of the model. Compared with the 6-parameter model, the calculation accuracy and correlation of this model have obvious advantages, and the calculated molecular absorption energy agrees well with the experimental values. At the same time, the calculation time does not consume more time and the efficiency is better.
2. The scalability of the model is better. In this paper, the performance of the model is confirmed by predicting the adsorption energy of the molecule. The computational model based on deep learning can be extended to the fields of calculation, optimization and prediction of various physics, chemistry, pharmacy and biology subjects. It has good scalability and can be used by the researchers in a lot of disciplines.
3. Although, from the perspective of prediction accuracy, we judge that the model proposed in this paper has no over-fitting phenomenon, in theory, the over-fitting problem of this model is unconfirmed.
4. The characteristic of the computational model is good accuracy and efficiency, but it also lacks the physical and chemical interpretation of theoretical calculations. For example, in this paper, how do the eight parameters and the weights affect the absorption energy? At the same time, whether there are other factors that have a greater impact on the calculation can not be reflected in this model.

## Conclusions

A novel CAPSO algorithm was proposed in this paper and applied for the optimization of the weight and deviation of DNNs. A prediction model abbreviated as CAPSO BP DNN was obtained. On the basis of a prediction experiment on molecular absorption energies, the CAPSO BP DNN model exhibited an outstanding performance in predicting molecular absorption energies with an accuracy and correlation obviously better than those of other algorithms. Because the essence of the DNN model is nonlinear network learning, the CAPSO BP DNN model can be inferred to merit the role of a reference in predicting other nonlinear chemical problems. The author will conduct in-depth research on extending the application field of the model and model establishing. In particular, how to use big data technology to solve calculation and modeling problems in the chemical field is worth profound discussion.

## Supplementary information

supplementary material
