# Supplementary material for: Prediction Model of Organic Molecular Absorption Energies based on Deep Learning trained by Chaos-enhanced Accelerated Evolutionary algorithm
Source: Sci Rep. 2019 Nov 21;9:17261. doi: 10.1038/s41598-019-53206-1 (PMC6872818; doi:10.1038/s41598-019-53206-1)
Supplement: Supplementary file 1 — supplementary material [file 41598_2019_53206_MOESM1_ESM.pdf]

Supplemental Information

Prediction Model of Organic Molecular Absorption Energies based on Deep Learning trained by Chaos-enhanced Accelerated Evolutionary algorithm

Mengshan Li, Suyun Lian, Fan Wang, Yanying Zhou, Bingsheng Chen, Lixin Guan and Yan Wu

S1. The datasets collected and the input/output data of the different models.

NO.	Experimental	GABP1	GABP2	LS-SVM	DP-DP-PSO RBF ANN	CAPSO BP DNN	Set
1	2.89	2.88	2.68	2.92	2.88	2.758042303	Testing
2	2.95	3.01	3.11	3.07	3.13	2.742886409	Testing
3	3.43	3.44	3.46	3.51	3.68	3.612860042	Testing
4	3.46	3.56	3.68	3.58	3.57	3.618037149	Testing
5	3.45	3.43	3.49	3.42	3.45	3.399020943	Testing
6	3.6	3.51	3.25	3.62	3.83	3.510340797	Testing
7	4.3	4.66	4.63	4.33	4.58	4.012268903	Testing
8	4.34	4.22	3.94	4.29	4.06	4.217010313	Testing
9	4.44	4.34	4.2	4.38	4.26	4.127855994	Testing
10	4.54	4.29	4.38	4.3	4.38	4.666565589	Testing
11	4.11	4.39	4.36	4.25	4.32	4.425546662	Testing
12	4.9	4.8	4.91	4.56	4.51	5.019316143	Testing

NO.	Experimental	GABP1	GABP2	LS-SVM	DP-DP-PSO RBF ANN	CAPSO BP DNN	Set
13	4.77	4.45	4.55	4.78	4.86	5.030298203	Testing
14	4.96	4.88	4.76	4.77	4.75	5.232139886	Testing
15	4.92	4.95	4.98	4.96	4.81	4.589869903	Testing
16	4.96	4.67	4.65	5.01	4.6	5.212249924	Testing
17	4.92	5.17	5.22	5.42	5.39	5.011840203	Testing
18	6.05	5.86	5.76	5.88	5.78	6.08743255	Testing
19	5.17	5.17	5.23	5.34	5.36	5.367728138	Testing
20	5.28	4.92	5.29	5.24	5.25	5.285580712	Testing
21	5.1	4.87	4.91	5.06	5.1	5.337443929	Testing
22	5.32	5.14	5.18	5.12	5.01	5.09272242	Testing
23	6.08	6.01	5.99	5.62	5.82	6.355107238	Testing
24	5.12	5.15	5.03	5.01	4.93	5.212571142	Testing
25	2.98	2.99	3.13	3.01	2.86	2.962490103	Training
26	2.99	2.99	3.09	3.04	2.92	2.938736682	Training
27	2.83	2.83	2.8	2.89	2.75	2.730800964	Training
28	2.71	3.04	2.87	2.86	2.58	2.822085196	Training
29	2.96	2.93	3.08	2.99	2.85	2.952848956	Training
30	2.69	2.78	2.71	2.68	2.76	2.695490322	Training
31	2.73	2.8	2.93	2.69	2.76	2.577966016	Training
32	2.88	2.98	2.85	2.89	2.86	2.733298938	Training
33	3.1	3.2	3.03	3.15	3.14	3.183484591	Training
34	3.84	3.94	4.05	4.11	3.92	3.964872134	Training
35	3.63	3.45	3.44	3.58	3.53	3.619170576	Training
36	3.05	3.13	2.97	3.11	3.05	3.229179839	Training

NO.	Experimental	GABP1	GABP2	LS-SVM	DP-DP-PSO RBF ANN	CAPSO BP DNN	Set
37	3.81	3.76	3.72	4	3.76	3.969146474	Training
38	3.09	3.03	3.13	3.03	3.15	3.149385005	Training
39	3.23	3.18	3.23	3.25	3.26	3.409361669	Training
40	3.21	3.17	3.32	3.21	3.33	3.048426252	Training
41	3.25	3.09	3.39	3.22	3.37	3.365505227	Training
42	3.96	4.16	4.19	3.59	3.85	4.0865677	Training
43	3.43	3.5	3.46	3.39	3.28	3.311589785	Training
44	3.43	3.4	3.24	3.33	3.37	3.348734427	Training
45	3.32	3.24	3.22	3.24	3.23	3.293635148	Training
46	3.09	3.06	3.06	2.95	3.05	3.259436805	Training
47	3.15	2.98	3.12	3.15	3.25	3.147917842	Training
48	3.01	3.03	3.14	3.02	3.04	3.131265195	Training
49	3.83	3.96	4.01	4.08	3.82	3.700275034	Training
50	3.78	3.94	3.71	3.59	3.88	3.767185396	Training
51	3.87	3.95	3.89	3.9	3.79	3.72532364	Training
52	3.76	3.63	3.43	3.95	3.66	3.866551234	Training
53	3.43	3.55	3.32	3.37	3.35	3.496722463	Training
54	3.91	4.02	3.87	3.68	3.83	4.059636416	Training
55	4.66	4.62	4.66	4.62	4.71	4.600435505	Training
56	4.43	4.7	4.71	4.54	4.58	4.496304559	Training
57	4.38	4.52	4.57	4.53	4.51	4.480880634	Training
58	4.32	4.32	4.39	4.26	4.33	4.131472601	Training
59	4.66	4.48	4.43	4.53	4.59	4.477478126	Training
60	4.22	4.29	4.31	4.2	4.46	4.087806878	Training

NO.	Experimental	GABP1	GABP2	LS-SVM	DP-DP-PSO RBF ANN	CAPSO BP DNN	Set
61	4.66	4.71	4.78	4.66	4.84	4.573915098	Training
62	4.49	4.55	4.58	4.5	4.43	4.365728765	Training
63	4.18	4.21	4.19	4.53	4.47	4.098821783	Training
64	4	4.06	4.24	3.94	4.22	4.043364723	Training
65	4.4	4.49	4.51	4.41	4.32	4.42056621	Training
66	4.66	4.82	4.78	4.49	4.61	4.849623724	Training
67	4.13	4.33	4.15	4.28	4.5	4.088143035	Training
68	4.11	4.21	4.11	4.1	4.14	4.06189124	Training
69	4.28	4.53	4.53	4.33	4.49	4.187965396	Training
70	4.46	4.39	4.43	4.69	4.66	4.655558225	Training
71	4.58	4.64	4.6	4.55	4.56	4.603546677	Training
72	4.4	4.69	4.74	4.44	4.48	4.469745373	Training
73	4.07	4.52	4.38	4.24	4.1	4.101501242	Training
74	4.68	4.75	4.78	4.53	4.47	4.849639331	Training
75	4.12	4.27	4.18	3.95	4.18	3.921943968	Training
76	4.53	4.99	4.87	4.54	4.74	4.579893586	Training
77	4.07	4.1	4.08	4.13	4.08	4.180092324	Training
78	4.7	4.83	4.86	4.77	4.72	4.710671532	Training
79	4.71	4.77	4.8	4.76	4.8	4.78630421	Training
80	5	4.86	4.94	5.01	4.95	5.168974034	Training
81	4.84	4.86	4.81	4.84	4.8	4.710797782	Training
82	4.92	4.81	4.8	4.76	4.72	4.844796012	Training
83	4.96	4.69	4.84	5.19	4.84	4.858308643	Training
84	5	5.03	5.11	4.8	4.91	4.88477885	Training

NO.	Experimental	GABP1	GABP2	LS-SVM	DP-DP-PSO RBF ANN	CAPSO BP DNN	Set
85	5.06	5.38	5.53	5.05	5.36	4.992014003	Training
86	4.92	4.84	4.9	5.11	4.99	4.868226526	Training
87	5	5.37	5.29	5.31	5.42	5.067261175	Training
88	4.81	4.56	4.69	4.76	4.86	4.753225543	Training
89	4.84	5.18	5.29	4.79	4.88	4.73696931	Training
90	4.77	4.77	4.81	4.65	4.69	4.829521752	Training
91	4.96	5.05	5.11	4.94	5.12	5.102075768	Training
92	4.75	5.17	5.35	4.74	5.01	4.77307151	Training
93	5.06	4.68	4.65	5.08	5.01	5.010272727	Training
94	4.96	4.97	5.52	4.83	5.14	5.068468301	Training
95	4.96	4.87	4.91	4.69	4.25	5.038688948	Training
96	5	5	4.61	4.85	4.75	4.953867305	Training
97	4.84	5.05	5.06	4.9	4.93	4.753672625	Training
98	5	4.94	4.99	4.78	4.83	4.935549549	Training
99	4.83	4.57	4.63	4.84	4.83	5.003764017	Training
100	5	4.87	4.88	5.1	5.05	5.199198988	Training
101	5.02	5.16	5.29	5.1	5.03	4.993220946	Training
102	4.9	4.9	4.9	4.81	4.78	4.935063731	Training
103	4.82	4.75	4.82	5.07	4.98	4.921278215	Training
104	5	4.78	4.84	4.89	4.92	4.82446341	Training
105	5.23	5.09	5.15	5.32	5.23	5.113004529	Training
106	5.63	5.48	5.86	5.67	5.41	5.816083026	Training
107	5.17	5.15	5.21	5.24	5.22	5.075487982	Training
108	5.15	5.34	5.38	5.12	5.14	5.262154101	Training

NO.	Experimental	GABP1	GABP2	LS-SVM	DP-DP-PSO RBF ANN	CAPSO BP DNN	Set
109	5.88	5.78	5.66	5.53	5.76	5.799696778	Training
110	5.47	5.67	5.52	5.44	5.53	5.34734986	Training
111	5.46	5.08	5.15	5.47	5.67	5.610493401	Training
112	5.12	5.11	5.46	5.29	5.52	5.282494653	Training
113	5.21	5.04	5.14	5.25	5.01	5.383429506	Training
114	5.99	5.99	5.98	5.93	5.8	5.923328864	Training
115	5.91	5.83	5.67	5.78	5.46	6.028712966	Training
116	5.19	5.17	5.25	5.05	5.12	5.179648169	Training
117	5.56	5.46	5.51	5.54	5.39	5.414073017	Training
118	5.12	4.75	4.83	5.14	4.81	5.263507757	Training
119	5.41	5.09	5.45	5.28	5.56	5.512278741	Training
120	5.74	5.79	5.64	5.88	5.79	5.616674207	Training
121	5.56	5.22	5.18	5.46	5.41	5.682916527	Training
122	5.1	5.06	5.05	5.11	5.17	5.275617061	Training
123	5.79	5.7	5.65	6.01	5.9	5.84808629	Training
124	5.17	5.08	5.19	4.95	4.98	5.316908093	Training
125	5.51	5.35	5.37	5.28	5.52	5.622536182	Training
126	5.12	5.01	5.06	5.18	5.01	5.272864075	Training
127	5.99	5.87	5.87	6.14	5.81	5.984935251	Training
128	5.39	5.69	5.67	5.21	5.53	5.47642291	Training
129	5.99	5.44	5.58	5.77	6.06	6.032911644	Training
130	6.05	5.58	5.45	5.58	5.52	6.195061901	Training
131	5.46	5.28	5.32	5.53	5.31	5.52006392	Training
132	5.32	4.8	4.82	5.45	5.27	5.150533226	Training

NO.	Experimental	GABP1	GABP2	LS-SVM	DP-DP-PSO RBF ANN	CAPSO BP DNN	Set
133	5.3	5.38	5.38	5.51	5.5	5.414602915	Training
134	5.58	5.62	6.05	5.61	5.89	5.735080092	Training
135	5.32	5.29	5.34	5.59	5.51	5.241013088	Training
136	5.39	5.28	5.25	5.24	5.23	5.319468555	Training
137	2.98	2.93	2.98	3.01	2.89	3.036255601	Validation
138	2.87	2.84	2.89	2.93	2.82	3.102531021	Validation
139	3.18	3.15	3.12	3.17	3.17	3.314851747	Validation
140	3.19	3.15	3.15	3.2	3.21	2.991564484	Validation
141	3.38	3.34	3.39	3.33	3.34	3.165987246	Validation
142	3.57	3.62	3.71	3.76	3.86	3.765548298	Validation
143	4.07	4.11	4.1	4.21	4.2	3.977009465	Validation
144	4.11	4.11	4.32	4.35	4.36	4.216648931	Validation
145	4.28	4.77	4.6	4.73	4.7	4.037620634	Validation
146	4.16	4.31	4.25	4.2	4.26	3.982592208	Validation
147	4.63	4.78	4.75	4.85	4.69	4.73124674	Validation
148	4.77	4.86	4.93	4.84	4.79	4.662228955	Validation
149	4.86	4.95	5.01	5.64	5.23	5.070445465	Validation
150	5.08	5.27	5.31	5.1	5.2	4.833609324	Validation
151	4.85	4.65	4.73	5.01	4.69	4.711158554	Validation
152	4.94	5.23	5.31	5.33	5.31	5.127217141	Validation
153	4.71	4.64	4.74	4.85	4.78	4.704615716	Validation
154	5.17	4.63	4.81	5.18	4.99	5.003362242	Validation
155	6.66	6.66	6.45	5.96	5.85	6.834573657	Validation
156	5.56	5.77	5.66	5.82	5.76	5.454451354	Validation

NO.	Experimental	GABP1	GABP2	LS-SVM	DP-DP-PSO RBF ANN	CAPSO BP DNN	Set
157	5.19	5.07	5.09	5.02	5.18	5.254119387	Validation
158	5.69	5.52	5.53	5.82	5.64	5.898860362	Validation
159	5.25	5.12	5.12	5.35	5	5.291697679	Validation
160	5.44	5.5	5.4	5.57	5.46	5.255657149	Validation

S2. The descriptors of 160 molecules.

NO.	theoretical calculating value Ec of absorption energy (B3LYP/STO-3G)	molecular electron number Ne --normalized	oscillator strength Os--normalized	number of double bonds Ndb --normalized	total number of atoms Na, --normalized	number of hydrogen atoms Nh --normalized	number of carbon atoms Nc, --normalized	number of nitrogen atoms NN--normalized
1	4.95	0.133333333	0.532888	0	0.466667	0.066667	0.533333	0.06666667
2	3.51	0.533333333	0.055881	0.533333	0.466667	0.733333	0.733333	0.26666667
3	3.18	0.8	0.240874	0.6	0.4	1	0.4	0
4	2.96	0.133333333	0.487392	0.333333	0.066667	0.6	0.333333	0.73333333
5	5.7	0.8	0.653173	0.733333	0.466667	0.133333	0.6	0.46666667
6	4.7	0.8	0.305134	0.466667	0.066667	0.933333	0.933333	0.73333333
7	3.83	0.133333333	0.485773	0.8	0.333333	0.8	0.333333	1
8	3.46	0	0.738743	0.6	0.666667	0	0.533333	0.13333333
9	5.58	0.8	0.707074	0.066667	0.533333	0.333333	0.533333	0.8
10	4.56	0.4	0.50688	0.133333	0.066667	0.266667	0.266667	0.4
11	4.01	0.333333333	0.816905	0.866667	1	0.333333	0.666667	1
12	5.74	0.333333333	0.875135	0	0.533333	0.933333	0.133333	0.6
13	4.16	0.733333333	0.988862	0.8	0.066667	0.2	0.533333	0.66666667
14	3.61	0.733333333	0.047742	1	0.333333	0.333333	0.133333	1
15	3.13	0.333333333	0.476074	0.666667	0.133333	0.666667	0.666667	0.4
16	4.25	0.666666667	0.623071	0.2	0.533333	0.866667	0.533333	0.53333333
17	3.63	0.133333333	0.925936	0.466667	0.533333	0.4	0.8	1
18	3.25	0	0.544344	0.066667	0.266667	0.466667	1	1

NO.	theoretical calculating value Ec of absorption energy (B3LYP/STO-3G)	molecular electron number Ne --normalized	oscillator strength Os--normalized	number of double bonds Ndb --normalized	total number of atoms Na, --normalized	number of hydrogen atoms Nh --normalized	number of carbon atoms Nc, --normalized	number of nitrogen atoms NN--normalized
19	2.99	0.666666667	0.859927	0.533333	0.666667	0.733333	0.6	0.6
20	8.27	0.2	0.625363	0.866667	0.2	0.733333	0.733333	0.73333333
21	5.89	0.466666667	0.022453	0.866667	0	0.2	0.333333	0
22	5.49	0.866666667	0.58697	0.4	0.2	0.4	0.533333	0.06666667
23	4.94	0.333333333	0.54517	0.133333	1	0.4	0.266667	0.33333333
24	4.66	0.066666667	0.472027	0.733333	0	0.4	0.2	0.66666667
25	4.52	0.466666667	0.400567	0.266667	0.266667	0.733333	0.866667	0.6
26	4.42	0.2	0.974698	0.933333	0.533333	0.6	0.866667	0.13333333
27	3.54	0.533333333	0.195798	0.533333	0.8	0.4	0.533333	0.73333333
28	5.21	0.333333333	0.600677	0.266667	0.8	0.266667	0.333333	0
29	4.69	0.866666667	0.744243	0.933333	0.6	0.733333	0.4	0.26666667
30	3.91	0.066666667	0.059183	0	0.733333	0.733333	0.2	0.4
31	4.03	0.133333333	0.003709	0.733333	0.133333	0.066667	1	0.53333333
32	3.69	0.933333333	0.743023	0.666667	0.733333	0.733333	0.533333	0.66666667
33	3.87	0.133333333	0.261111	0.6	0.066667	0.8	0.866667	0.46666667
34	3.88	0.866666667	0.422658	0.533333	0	0.4	0.133333	0.46666667
35	3.3	0.133333333	0.682169	1	0.466667	0	0.266667	0.2
36	3.12	0.8	0.06924	0.533333	0.733333	0.666667	0.333333	0.33333333
37	2.89	0.4	0.530855	0.133333	0.6	0.4	0.666667	0
38	3.34	0.466666667	0.231603	0	0	0.8	0.533333	0.6

NO.	theoretical calculating value Ec of absorption energy (B3LYP/STO-3G)	molecular electron number Ne --normalized	oscillator strength Os--normalized	number of double bonds Ndb --normalized	total number of atoms Na, --normalized	number of hydrogen atoms Nh --normalized	number of carbon atoms Nc, --normalized	number of nitrogen atoms NN--normalized
39	3.22	0.266666667	0.580481	0.466667	0.933333	0.333333	0.666667	0.6
40	2.81	0.266666667	0.660902	0.066667	0.8	0.066667	0.4	0.13333333
41	4.51	0.6	0.650598	0.4	0.533333	0.533333	0.533333	1
42	3.95	0.2	0.930163	1	0.4	0.8	0.266667	0.4
43	3.47	0.8	0.610699	1	0.733333	0.333333	0.266667	0.06666667
44	3.17	0.4	0.425109	0.6	0.466667	0.466667	0.466667	0.26666667
45	4.49	0.666666667	1	0.266667	0.6	0.2	0.266667	0
46	3.84	0.933333333	0.495258	0.2	0.133333	0.4	0.466667	0.33333333
47	3.25	0.6	0.854377	0.6	0.333333	1	1	0.26666667
48	3.36	0.666666667	0.801576	0.666667	0.2	0.666667	0.2	0.26666667
49	3.09	0.2	0.528936	0.6	0.4	0.133333	0.933333	0.06666667
50	3.36	0.733333333	0.28994	0.866667	0.533333	0.866667	0.866667	0.26666667
51	2.97	0.266666667	0.060469	0.466667	0.6	0.266667	0.733333	0.66666667
52	7.18	0.8	0.109743	0.6	0.2	0.733333	0.066667	0.86666667
53	7.16	0.333333333	0.032584	0.066667	0.533333	0.933333	0.733333	0.66666667
54	5.54	0.533333333	0.04627	0.866667	0.133333	0.8	0.933333	0.26666667
55	5.65	0.733333333	0.743705	0.266667	0.133333	0.8	0.4	1
56	7.38	0.866666667	0.773067	0.733333	0	0.333333	0.533333	0.26666667
57	5.31	0.333333333	0.704815	0	0	0.2	0.2	0.86666667
58	5.78	0.133333333	0.955127	0.533333	0.6	0.4	0.066667	0.53333333

NO.	theoretical calculating value Ec of absorption energy (B3LYP/STO-3G)	molecular electron number Ne --normalized	oscillator strength Os--normalized	number of double bonds Ndb --normalized	total number of atoms Na, --normalized	number of hydrogen atoms Nh --normalized	number of carbon atoms Nc, --normalized	number of nitrogen atoms NN--normalized
59	6.2	0.533333333	0	0.4	0.6	0.333333	0.8	0.33333333
60	6.19	0.8	0.369158	0.666667	0.866667	0.066667	0.866667	0.33333333
61	5.52	0.466666667	0.540974	0.933333	0.733333	0.133333	0.533333	0.6
62	7.08	0.6	0.982542	0.866667	0.133333	0.466667	0.266667	0.86666667
63	6.05	0.533333333	0.866221	1	0.4	0.266667	0.266667	0.13333333
64	7.67	0.4	0.863899	0.866667	0.133333	0.066667	0.866667	0.8
65	6.52	0.733333333	0.528041	0.133333	0.6	0.533333	0.466667	0.8
66	6.18	0.933333333	0.302797	0.8	0.533333	0.2	1	0.53333333
67	5.28	0.333333333	0.454444	0.6	0.066667	0.933333	0	1
68	5.03	0.866666667	0.016854	0.466667	0.6	0.133333	0.266667	0.26666667
69	6.31	0.2	0.698259	0.2	1	0.8	0.533333	0.13333333
70	6.19	0.2	0.060173	0	0.2	0.2	0.066667	0
71	5.74	0.266666667	0.16003	0	0.866667	0.133333	1	0.4
72	6.52	0.666666667	0.168976	0.133333	0.466667	0.466667	0.2	0.53333333
73	6.49	0.6	0.7683	0	0	0.666667	0.6	0
74	7.09	0.533333333	0.2178	0.666667	0.933333	0.2	0.333333	0.33333333
75	5.73	0.466666667	0.424069	0.733333	0.133333	0.533333	0.2	0.33333333
76	6.98	0.866666667	0.10481	1	0.466667	0.666667	0.333333	0.46666667
77	5.88	0.933333333	0.026752	0.866667	0.333333	0.2	0.466667	0.86666667
78	6.47	0.733333333	0.859709	0.8	0.6	0.533333	1	0.33333333

NO.	theoretical calculating value Ec of absorption energy (B3LYP/STO-3G)	molecular electron number Ne --normalized	oscillator strength Os--normalized	number of double bonds Ndb --normalized	total number of atoms Na, --normalized	number of hydrogen atoms Nh --normalized	number of carbon atoms Nc, --normalized	number of nitrogen atoms NN--normalized
79	6.41	0.533333333	0.024906	1	0.866667	0	0.866667	0.4
80	6.11	0.466666667	0.195898	1	0.333333	0.4	0.733333	0.53333333
81	6.4	0.2	0.974456	0.6	0.666667	0.066667	1	0.06666667
82	5.3	0.2	0.124909	0.466667	0.666667	0.933333	0.533333	0.33333333
83	6.28	0.333333333	0.115511	0.666667	0.466667	0.333333	1	1
84	6.07	0.733333333	0.372076	0.4	0.6	0.2	0	0.93333333
85	5.96	0.2	0.504242	0.4	0.866667	0.733333	0.666667	0.4
86	6.16	0.4	0.66764	0.6	0.933333	0.4	0.333333	0.66666667
87	6.2	0.8	0.353579	0.666667	0.466667	0.266667	0.6	0.26666667
88	6.32	0.8	0.388257	0.066667	0.8	0.066667	0.733333	0.73333333
89	7.06	0.066666667	0.031165	1	0.666667	0.533333	0.133333	0.06666667
90	6.03	0.866666667	0.313513	0.8	0.066667	0.8	0.133333	0.2
91	6.8	0.733333333	0.049021	0.466667	0.733333	0.4	0.466667	0.93333333
92	7.17	0.933333333	0.56125	0.333333	0.4	0.466667	0.533333	0.2
93	6.75	0.266666667	0.810917	0.333333	0.2	0.266667	0.666667	0.06666667
94	7.38	0.733333333	0.150954	0.8	0.466667	0.533333	0.733333	0.26666667
95	7.33	0	0.691469	0.533333	0.533333	0.266667	0	1
96	6.98	0.466666667	0.019841	0.8	0.066667	1	0.4	0.6
97	5.61	0	0.465756	0	0.133333	0	0.533333	1
98	6.7	0.333333333	0.01144	1	1	1	0.2	0.46666667

NO.	theoretical calculating value Ec of absorption energy (B3LYP/STO-3G)	molecular electron number Ne --normalized	oscillator strength Os--normalized	number of double bonds Ndb --normalized	total number of atoms Na, --normalized	number of hydrogen atoms Nh --normalized	number of carbon atoms Nc, --normalized	number of nitrogen atoms NN--normalized
99	6.1	0.733333333	0.688506	0.533333	0.8	0.733333	0.8	0.2
100	6.24	0.4	0.266508	0.866667	1	0.066667	0.866667	0.26666667
101	6.1	0.066666667	0.341279	0.133333	0.933333	0.266667	0.266667	0.93333333
102	5.99	0.266666667	0.447897	0.866667	0.133333	0.066667	1	0.66666667
103	6.11	0.6	0.922768	0	0.533333	0.333333	0	0.66666667
104	6.37	0.6	0.270006	0.266667	0.666667	0.533333	0.133333	0.6
105	6.47	0.4	0.873123	0.6	0.866667	0.2	0.6	0
106	6.24	1	0.226631	0.066667	0.266667	0.6	0.533333	0.73333333
107	6.37	0.066666667	0.198585	0.4	0.533333	0	0.133333	0.4
108	7.06	0.2	0.483866	0.4	0.133333	0.733333	0.133333	0.66666667
109	5.49	0.333333333	0.940402	0.466667	0.8	0.8	0	1
110	5.78	1	0.302397	0.733333	0.533333	0.266667	0.333333	0.93333333
111	5.04	0.2	0.66242	0.066667	0.866667	0.733333	0.133333	0.13333333
112	6.49	0.4	0.283247	0.066667	0.533333	0.866667	0	0.33333333
113	5.87	0.933333333	0.998752	0.266667	0.8	0.333333	1	0.66666667
114	5.64	0.533333333	0.218141	0.6	0.8	0	0.066667	0.2
115	6.14	0.666666667	0.170587	0	0.133333	0.466667	1	0.26666667
116	4.79	0.2	0.042893	0.533333	0.8	1	0.866667	0.93333333
117	4.27	0.133333333	0.031516	0.866667	0.333333	0.333333	0.533333	0.33333333
118	4	0.133333333	0.30948	0.4	0.8	0.2	0.266667	0.53333333

NO.	theoretical calculating value Ec of absorption energy (B3LYP/STO-3G)	molecular electron number Ne --normalized	oscillator strength Os--normalized	number of double bonds Ndb --normalized	total number of atoms Na, --normalized	number of hydrogen atoms Nh --normalized	number of carbon atoms Nc, --normalized	number of nitrogen atoms NN--normalized
119	4.66	0.533333333	0.756481	0.333333	0.6	0.066667	0.733333	0.93333333
120	4.99	0.4	0.706207	0.8	0.6	1	0.2	0.46666667
121	4.69	0.6	0.620938	0.333333	0.466667	0.6	0.8	0.2
122	5.03	0.066666667	0.097733	0.866667	0.533333	0.2	0.2	0.13333333
123	5.09	0.133333333	0.505897	0.333333	0.666667	0.6	0.933333	0.46666667
124	5.25	0.866666667	0.436465	0.666667	0.733333	0.2	0	0.86666667
125	4.79	0.8	0.507824	0.533333	0.866667	0.266667	0.6	0.06666667
126	4.92	1	0.488121	0.133333	0.533333	0.866667	1	0.8
127	5.38	0	0.016769	0.666667	1	0.933333	0.4	0
128	5.81	0.2	0.263099	0.466667	0.333333	0.466667	0.266667	0.4
129	4.97	0.066666667	0.596213	0.866667	0.866667	0.933333	0	0.33333333
130	5.52	0.533333333	0.290999	0	0	0.533333	0.933333	0.26666667
131	5.27	0.333333333	0.602861	0.2	0.2	0.866667	0	0.8
132	5.41	0.666666667	0.697898	0	0.533333	0	0.266667	0.2
133	5.47	0.666666667	0.910935	0.066667	1	0.533333	0.066667	0.53333333
134	5.5	0.666666667	0.09612	0.6	0.533333	0.533333	0.333333	1
135	5.34	0.866666667	0.515855	0.6	1	0.133333	0.4	0.26666667
136	5.26	0.733333333	0.139514	1	0.733333	0.533333	0.6	0.33333333
137	5.44	0.133333333	0.926913	0.133333	0.733333	1	0.133333	0.66666667
138	5.11	1	0.479512	0.133333	0.4	0.2	0.866667	0.46666667

NO.	theoretical calculating value Ec of absorption energy (B3LYP/STO-3G)	molecular electron number Ne --normalized	oscillator strength Os--normalized	number of double bonds Ndb --normalized	total number of atoms Na, --normalized	number of hydrogen atoms Nh --normalized	number of carbon atoms Nc, --normalized	number of nitrogen atoms NN--normalized
139	5.57	0.333333333	0.158887	0.2	0.066667	0.733333	0.466667	1
140	5.2	0.066666667	0.099437	0.733333	0	0.733333	0.2	0.86666667
141	5.45	0.866666667	3.66E-06	0.133333	0.266667	0.533333	1	0.93333333
142	5.62	0.133333333	0.405503	0	0.866667	0	0.4	0.2
143	5.79	0.666666667	0.075036	0.866667	0.733333	0.666667	0.466667	0.8
144	5.74	0.933333333	0.730382	0.666667	0.866667	0.933333	0	0.6
145	5.75	0.133333333	0.43996	1	0.8	0.066667	0.6	0.8
146	6.1	0.2	0.422006	0.133333	0.066667	0.533333	0	1
147	5.53	0.4	0.734912	0.066667	0.4	0.333333	0.333333	0.8
148	6.01	0.333333333	0.925707	1	0.8	0.333333	0.8	0.8
149	6.16	0.6	0.661178	0.6	0.333333	0.066667	0.066667	0.6
150	5.4	0.666666667	0.335003	0	0.666667	0.266667	0.866667	0.13333333
151	6.82	0.533333333	0.945425	0	0.6	0.733333	0.533333	0.8
152	8.06	0.533333333	0.048297	0.933333	1	0.533333	0.266667	1
153	5.9	0.066666667	0.643029	0.533333	0.533333	0.6	0.6	0.26666667
154	5.9	0.6	0.538268	0.066667	0.733333	0.933333	0.666667	0.6
155	6.85	0.8	0.033982	0.2	0.066667	0.066667	0	0.33333333
156	5.83	0.666666667	0.339521	0.8	0.066667	0.8	0.066667	0.2
157	7.2	0.066666667	0.100588	0.8	0.733333	0.533333	0.933333	0.6
158	5.69	0.6	0.85055	0.133333	0.333333	0.866667	0.2	0.46666667

NO.	theoretical calculating value Ec of absorption energy (B3LYP/STO-3G)	molecular electron number Ne --normalized	oscillator strength Os--normalized	number of double bonds Ndb --normalized	total number of atoms Na, --normalized	number of hydrogen atoms Nh --normalized	number of carbon atoms Nc, --normalized	number of nitrogen atoms NN--normalized
159	6.32	0.533333333	0.280661	0.466667	0.6	0.2	0.066667	0.06666667
160	5.4	0.333333333	0.81623	0.466667	0.666667	0.4	0.866667	0.4
